# *In Vitro* Disease Models for Understanding Psoriasis and Atopic Dermatitis

**DOI:** 10.3389/fbioe.2022.803218

**Published:** 2022-02-21

**Authors:** Roudin Sarama, Priya K. Matharu, Yousef Abduldaiem, Mab P. Corrêa, Cristiane D. Gil, Karin V. Greco

**Affiliations:** ^1^ Research and Development Department, The Griffin Institute, Harrow, United Kingdom; ^2^ Division of Surgery and Interventional Science, University College London (UCL), London, United Kingdom; ^3^ Programa de Pós-Graduação Em Biociências, Instituto de Biociências Letras e Ciências Exatas, Universidade Estadual Paulista (UNESP), São José, Brazil; ^4^ Departamento de Morfologia e Genética, Escola Paulista de Medicina, Universidade Federal de São Paulo (UNIFESP), São José, Brazil

**Keywords:** psoriais, keratinocyte, fibroblast, *in vitro*, 3D models, atopic dermatitis (AD)

## Abstract

Psoriasis (PS) and Atopic Dermatitis (AD) are two of the most prevalent inflammatory skin diseases. Dysregulations in the immune response are believed to play a crucial role in the pathogenesis of these conditions. Various parallels can be drawn between the two disorders, as they are both genetically mediated, and characterised by dry, scaly skin caused by abnormal proliferation of epidermal keratinocytes. The use of *in vitro* disease models has become an increasingly popular method to study PS and AD due to the high reproducibility and accuracy in recapitulating the pathogenesis of these conditions. However, due to the extensive range of *in vitro* models available and the majority of these being at early stages of production, areas of development are needed. This review summarises the key features of PS and AD, the different types of *in vitro* models available to study their pathophysiology and evaluating their efficacy in addition to discussing future research opportunities.

## 1 Introduction

**FIGURE 1:**
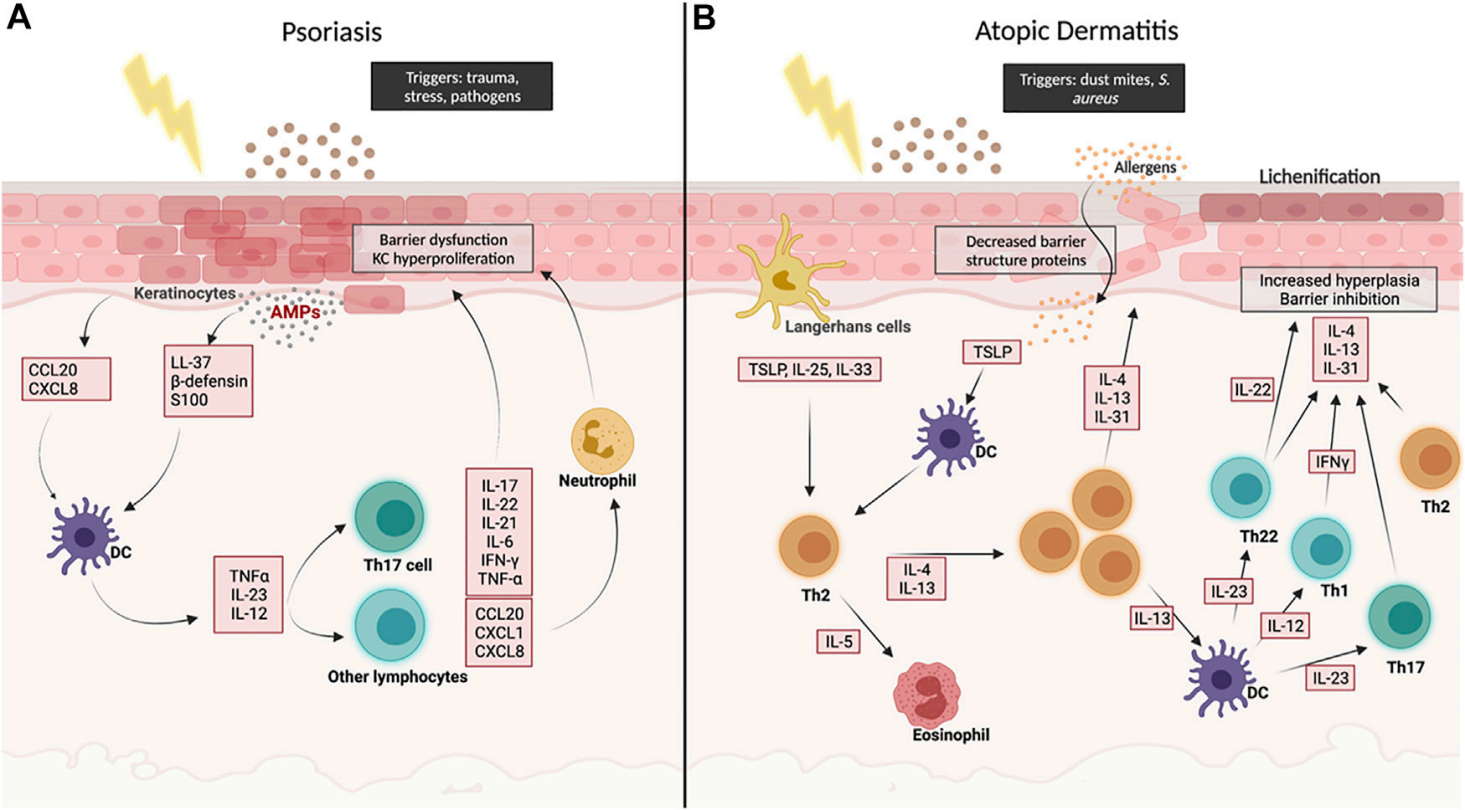
Schematic overview of the main molecular pathways involved in Psoriasis **(A)** and in Atopic Dermatitis **(B)**. Created with BioRender.com.

**FIGURE 2:**
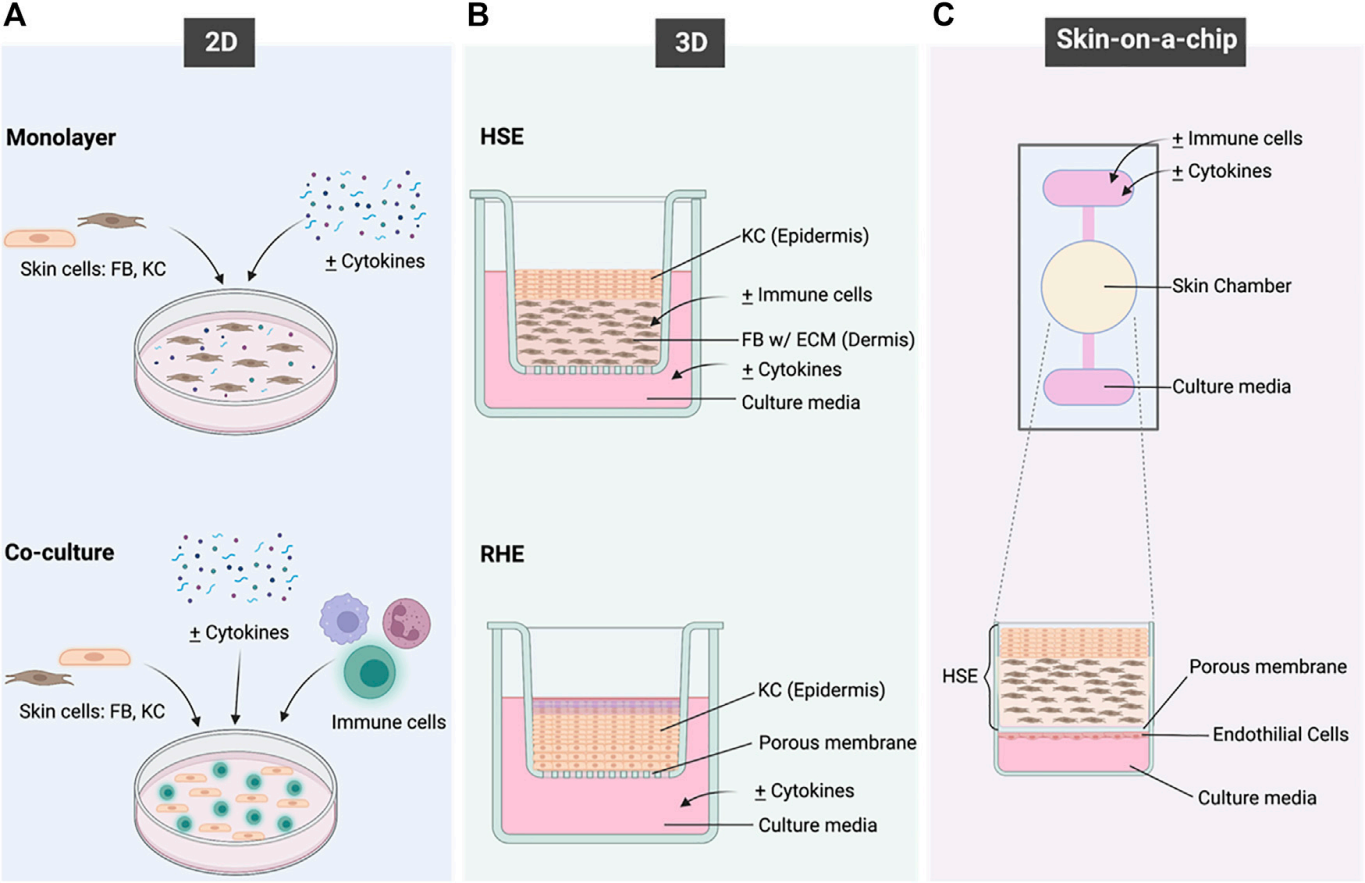
A schematic overview of the *in vitro* models that have been applied to recapitulate the immunological functions of the skin and to model inflammatory skin diseases. **(A)** shows the 2 different types of 2D models; **(B)** shows the HSE and RHE types of 3D models and **(C)** shows the concept of Skin-on-a-chip. Created with BioRender.com.

As one of the primary physical and immune barriers, skin is composed of the three principal layers: epidermis—the outermost layer; dermis—papillary and reticular regions and vascularized with blood vessels and nerves, and the hypodermis—the innermost layer. Epidermis is composed of several layers of keratinocytes (KC) and resident immune cells, including Langerhans cells and T lymphocytes (Bocheńska et al., 2017; Dainichi et al., 2018). KC can recruit and activate immune cells, resulting in the release of cytokines and chemokines that can affect proliferation triggering an inflammatory cascade (Dainichi et al., 2018). When the protection mechanisms of the skin become dysfunctional, several types of inflammatory diseases can be developed. Both Psoriasis (PS) and Atopic Dermatitis (AD) are chronic inflammatory diseases driven mainly by T-cells and have complex pathophysiology that need investigation (Figure 1). In order to fully understand the inflammatory process underlying these diseases, 2D monolayer and co-culture models (Figure 2A), 3D skin models (Figure 2B) and skin-on-a-chip systems (Figure 2C) were developed.

### 1.1 Psoriasis

PS is an immune-mediated, chronic inflammatory skin disease that affects skin and nails, with genetic, environmental, and immunological factors playing a huge role in pathogenesis and disease progression. PS has an average global prevalence of around 11%, with psoriasis vulgaris being the most prevalent variant (Michalek et al., 2017). Generally, it manifests as raised, well demarcated plaques with scaling. Histologically, psoriatic lesions develop by premature hyperproliferative KC activity resulting from sustained inflammation causing acanthosis, hyperkeratosis, disruption of epidermal layers anatomy, and presence or absence of specific histological biomarkers (Rendon et al., 2019). Recent research shows the complexity of the pathogenesis of PS, however, there are still gaps that need to be filled. This lack of knowledge can be seen in the limitations of current treatment options, and the varying treatment effectiveness and toxicity between patients.

#### 1.1.1 Pathogenesis and Biomarkers

In order to evaluate which *in vitro* models can best recapitulate PS, foundational knowledge of pathogenesis and biomarkers is needed. While usually triggered by infection or trauma, PS is maintained by disturbances in the innate and adaptive immune responses in patients with environmental and genetic predispositions. Activation of dendritic cells (DCs) is a key factor in developing plaques in psoriasis. After DC activation, they undergo maturation and then travel to the lymph nodes and cause cell mediated responses of T cells (TH1, TH17, and TH22) through the release of Tissue Necrosis Factor-alpha (TNF-α), in addition to Interleukin (IL)-13, and IL-23 which induce TH1 and TH17 responses, respectively. Both pathways cause the release of IL-17 family (A-F) and other cytokines (IL-20, IL-22) and chemokines (CCL20, CXCL1, CXCL8) from T cells, neutrophils, and other cells (Figure 1) (Chiricozzi et al., 2018). The granular layer in PS is either diminished or considerably reduced compared to healthy skin. Additionally, the expression of late differentiation genes can be disturbed by the chronic inflammation, like cytokeratin 10 (CK10), which is expressed in the suprabasal layer of healthy skin but reduced or absent in psoriatic lesions (Tjabringa et al., 2008).

KI-67 antigen expression, a marker for proliferative process, is dramatically increased in acanthotic PS skin, in addition to Cytokeratin 16 (CK 16), which represents hyperproliferation and abnormal differentiation (Tjabringa et al., 2008; Desmet et al., 2017). Moreover, the expression of antimicrobial proteins (AMPs), which are thought to be involved in DC activation, such as beta-defensins, anti-leukoprotease (SKALP/ELAFIN), and S100A7 (Psoriasin) are strongly induced in psoriatic lesions and help in differentiating psoriatic skin from other inflammatory skin lesions (Chiricozzi et al., 2018). Acanthosis refers to epidermal thickening caused by rapid KC turnover. In healthy skin, it takes around 30 days for KCs to migrate from the basal layer to the surface of the epidermis, while in psoriasis, it only takes 6–8 days. Parakeratosis, which is the retention of nuclei in cornified layer caused by premature differentiation, is also common (Charifa et al., 2021).

### 1.2 Atopic Dermatitis

Atopic Dermatitis (AD) is one of the most common allergy-mediated inflammatory skin diseases, and the most common form of childhood eczema. Recent studies have shown AD affects around 23% of the global population, with up to 20% being children (Nutten, 2015). It is thought that a range of genetic and environmental factors contribute to the severity of pruritus from AD, and therefore treatments can vary significantly. Unlike other allergic conditions, AD flare-ups are manifested by a combination of complex immunological and predisposing factors. Typical characteristics of AD include chronic or the sudden onset of dry skin and intense pruritus, which when aggravated can lead to severe skin lesions (Peng and Novak, 2015).

#### 1.2.1 Pathogenesis and Biomarkers

Acute AD is triggered by the dual increase of CD4+ and T-Helper2 (Th2) cells in the epithelium. Th2 cells secrete a number of cytokines, including IL-4, IL-33, and IL-13, aiding in the promotion of Immunoglobulin-E (IgE) which is linked to causing hypersensitivity to allergens and impaired barrier function (Chiricozzi et al., 2020).

Where lesions are present it is common for the patient to suffer from infection caused by *staphylococcus aureus* present on the skin, entering the wound. In this case, Th2 cells and mast cells produce the pro-inflammatory cytokine, IL-31 triggering an immune response in reaction to the bacteria. The presence of IL-33 also produces other immune cells, including mast cells, neutrophils, and eosinophils in response to the infection, causing further inflammation and thus increased pruritus (Schmitz et al., 2005).

In chronic AD-associated lesions, decreased levels of IL-4 and IL-13 are observed, however, Th1 pro-inflammatory cytokines IL-5 and IL-12, and IFN-γ, which promote eosinophils and monocytes recruitment, are increased (Figure 1). This increase within the epidermis is thought to be the main cause of AD (Fiset et al., 2006).

Filaggrin is a protein encoded by the FLG gene that binds to keratin within the epithelium (Sandilands et al., 2009). Mutations within FLG have been identified as a common cause of Ichthyosis Vulgaris, a skin conditions where skin loses its ability to shed dead skin cells, leaving excess layers of dry and scaly skin. The inhibited production of filaggrin leads to the malformation of the epidermis and increases the chances of water loss, causing dry skin (Sandilands et al., 2009). Increased Thymic Stromal Lymphopoietin (TSLP) has also been found to be overexpressed in AD lesions (Tatsuno et al., 2015). The constant friction can lead to spongiosis (intracellular oedema within the epidermis) and lichenification of the skin; thick and leathery texture of skin, leading to KC hyperproliferation (hyperkeratosis) and parakeratosis (Guttman-Yassky et al., 2011).

AD and PS share similarities predominantly surrounding clinical presentations and triggers, but also several differences. For example, microarray analysis showed decreased levels of immune genes and cytokines compared to healthy skin (Nomura et al., 2003). Additionally, studies have shown that patients with AD frequently experience skin infections, due to the invasion of *Staphylococcus aureus* into the epidermis, however this is not common in PS (Nomura et al., 2003).

Due to the complex pathogeneses of both conditions, reproducing an accurate representation in a single *in vitro* system poses its challenges, however, several studies have developed models mimicking at least one aspect of the pathology, which is imperative when understanding the diseases.

## 2 *In vitro* Models

### 2.1 2D Cell Cultures

2D cell cultures are the simplest *in vitro* disease models for PS and AD, as they consist of either one type of cell in a monolayer or a co-culture of multiple cell types (Figure 2A). This culture system disregards complex structures and interactions within tissues but allows the evaluation of cell responses towards any given stimulus and understanding cell-to-cell interactions.

#### 2.1.1 Monolayer Models

Monolayer cultures of KC have been widely used for biological and pharmacological screening of anti-psoriatic drugs as they provide an easily reproducible model that remains useful for studying molecular pathways. The first attempt of creating a PS-2D-monolayer model was achieved by isolating psoriatic KC from lesioned psoriatic skin of patients. Unfortunately, this method was insufficient as the models lost the phenotype gene expression of cyclic-adenosine monophosphate (cAMP), Defensin Beta-4, PI3, and Tissue Necrosis Factor (TNF), and suffered cells’ growth potential limitations (Leigh et al., 1995). Recently, an inflammatory KC model was established using immortalised human KC (HaCaTs) in order to produce psoriasiform KC at the transcription level. The study identified a simulation mixture of proinflammatory cytokines (IL-17A, IL-22, IL-1α, TNF-α, and OSM) that induces inflammatory KC and shown to be beneficial in identifying potential PS biomarkers (Zheng et al., 2021).

Desmet and colleagues developed a drug-screening model for PS by adding foetal calf serum and inflammatory cytokines to the culture medium of healthy human KC and studied the expression of PS related genes and proteins. They found that the model had an increased expression of PS-related genes including Keratin-16 (K16), SKALP/ELAFIN, and Psoriasin, and proved to be advantageous for screening therapeutic drugs such as tofacinib (Desmet et al., 2017).

Similarly, monolayer models have been useful for understanding the underlying mechanism of AD. They are easily generated by culturing either HaCaT cells or primary human KC with IL-4 and IL-13, leading to the downregulation of the genes Krt1, Krt10, Dsg1 and Dsc1 as well as an increase in the fragmentation of the cell sheets alongside mechanical stress (Omori-Miyake et al., 2014). In addition, IL-4 and IL-13 resulted in decreased expression of filaggrin, consistent with AD characteristics (Howell et al., 2009). Another AD monolayer model was composed of CD4+ T cells derived from AD patients, with the aim to explore their correlation with TSLP. Lymphocytes were used in a monolayer model to explore the role that thymic stromal lymphopoietin has on T cells in AD cells. The study showed that T cells from AD patients have a strong ability to interact with TSLP which was found to upregulate IL-4 production, suggesting a positive feedback loop to maintain a persistent Th2 response (Tatsuno et al., 2015).

#### 2.1.2 Co-Culture Model

Although monolayer models are quite simple and beneficial in screening of anti-psoriatic drugs, they are limited by the absence of epidermal stratification. Saiag and colleagues studied the effect of psoriatic and healthy FBs on normal and psoriatic KCs. The authors concluded that psoriatic FB induce normal KC hyperproliferation, however, psoriatic KC remained hyperproliferative even with healthy FB, demonstrating that cell-cell interaction has an effect on the presentation of the disease, and that FBs have lower impact on the development of PS compared to KC (Saiag et al., 1985).

A second study focusing on the effect of co-culturing healthy or psoriatic KC with healthy T lymphocytes found that psoriatic KC enhanced T lymphocyte survival when co-cultured, with an overproduction of proinflammatory cytokines including TNF-α, IL-6, IL-8, GM-CSF, MCP-1 and IL-10. It also showed that in order to achieve cross-talking between cells, a direct cell-cell interaction must occur (Martin et al., 2012). Another study, however, demonstrated that direct cell-cell interaction was not necessary for crosstalk between T lymphocytes and KCs in their *in vitro* 3D model (van den Bogaard et al., 2014).

Co-culturing human dermal FBs with eosinophils/basophils was successfully used to understand the cytokine/chemokine release in AD. Co-culturing with basophils resulted in a strong release of CXCL8, CCL2, and CCL5, while eosinophil co-culturing resulted in increased release of IL-6, CXCL8, and CCL4 (Jiao et al., 2016). A recent study used activated T cells in a HaCaT-based model which expressed several AD biomarkers, including KC apoptosis, increased levels of pro-inflammatory cytokines/chemokines, and the increased expression of neutrotrophin-4 which is linked to pruritus surrounding skin lesions (Engelhart et al., 2005).

### 2.2 3D Cell Cultures

3D models have proven to be an advantageous alternative in tackling the limitations of monolayer cultures. To achieve an efficient epidermal barrier, keratinocytes must be exposed at the air–liquid interface and cultured in conditions that favour their stratification. 3D models for PS and AD can be divided into: Reconstructed Human Epidermis (RHE), and Human Skin Equivalent (HSE), which can be partial thickness (PT), or full thickness (FT) containing both epidermis and dermis as seen in Figure 2B (Desmet et al., 2017). Four types of matrices are used in developing skin equivalents:

#### 2.2.1 Porous Membrane

Porous membranes are used in PS models by co-culturing KCs and FBs on either side of the membrane, allowing intracellular interactions. A 1990 study showed that the presence of psoriatic FB on one side of the membrane leads to healthy KC on the other side to develop psoriasiform phenotype, validating the principle of cross-talk (Krueger and Jorgensen, 1990).

#### 2.2.2 Fibroblast-Containing Protein Scaffolds

These dermis-like scaffold are composed of a collagen and FB mixture, proving advantageous compared to synthetic polymers due to improved biocompatibility and cellular adhesion (Parenteau-Bareil et al., 2010). However, these scaffolds maintain a limited shelf-life and poor mechanical properties (Supp and Boyce, 2005).

#### 2.2.3 De-Epidermised Dermis

This model is formed by applying high temperatures to skin samples, followed by removing the epidermis, and was developed to overcome shortcomings faced by other matrices, such as the lack of skin morphology and the absence of cell-cell interactions. This model is beneficial because it can withstand culturing for up to 4–5 weeks. However, since the DED model lacks any viable fibroblasts, the extracellular membrane cannot self-renew or maintain growth factor accumulation (Desmet et al., 2017). Additionally, the need for excessive skin biopsies can also be seen as impracticable in high throughput drug screenings.

#### 2.2.4 Self-Assembly Model

This model allows dermal FB to retain the ability to produce, release, and organize their own extracellular matrix (ECM) without needing additional exogenous material or skin biopsies (Saba et al., 2018). Fibroblasts are cultured on a plastic platform with growth media supplemented with serum and ascorbic acid, until substantial proliferation has formed functioning tissue and native human ECM. After approximately 28 days, the fibroblasts are cultured into an ECM-like sheet, which can be layered to mimic a dermal sheet. KC are then seeded onto the dermal sheets, and the FT reconstruction is cultured at the air-liquid interface, promoting KC differentiation and epidermal formation (Auxenfans et al., 2009).

The use of PS patient derived skin cells to form FT-HSE is crucial to understand biocompatibility and interactions between KC and FB (Jean et al., 2009). FLG knockout KC were used in an HSE model to investigate skin barrier function in AD. The study did not observe barrier dysfunction, however the authors suggest that such dysfunctions are due to microbiome alterations along genetic mutations (Niehues et al., 2017).

3D models developed for PS and AD generally have similar features, due to their similar pathogenesis. An HSE model was generated by seeding a decellularized DED with human primary KC, followed by adding allogenic T cells underneath the dermis 7 days post culture. KC activation was seen within 2 days of T cell migration into the dermis, and an inflammatory response was evident after 4 days. This, along with the upregulation of epidermal PS and AD genes, increased the expression of pro-inflammatory cytokines and chemokines leading to decreased filaggrin and significant keratinocyte differentiation within the epidermis. Researchers concluded that this model was closer to resembling psoriasis, as it lacked important AD markers and morphological characteristics, such as spongiosis and apoptosis (van den Bogaard et al., 2014). Mutations in the FLG2 gene have been heavily associated with the downregulation of filaggrin-2 in the epidermis of AD patients. Pendaries and colleagues produced a 3D-RHE model for AD incorporating a decreased amount of filaggrin-2 using lentivirus-mediated shRNA, which resulted in parakeratosis, development of a compact stratum corneum, and presence of abnormal vesicles (Pendaries et al., 2015).

Interactions between KC and FB were studied using multiple FT-skin equivalents with different combinations of both healthy and Psoriatic KCs, and FBs on a collagen-based PS model. The psoriatic KC and FB demonstrated a higher proliferation rate, and increased expression of proinflammatory cytokines including TNF-alpha, INF-gamma, and IL-8 (Barker et al., 2004). Similarly, another study compared four different skin models by combining healthy and psoriatic cells. The model developed using psoriatic-KC presented increased epidermal thickness, higher cellular proliferation, and reduction of flaggirin, locirin, and K16. Additionally, histological analysis showed thickening of the epidermal layer, consistent with PS phenotype *in vivo* (Jean et al., 2009). Another psoriatic model was developed by adding TNF-α, IL-1α, IL-6, and IL17A on an HSE model with PS patient derived KC. This showed increased expression of S100A12, IL-8, DEFB4A, and KYNU (Pouliot-Bérubé et al., 2016). Even though using patient cells proved to be beneficial in the development of psoriasis, it is not widely used due to the scarcity of diseased cells, and heterogeneity of patient derived cells.

### 2.3 Skin-On-A-Chip

Skin-on-a-chip is a relatively new technology developed as an alternative to the current models by utilizing the concepts of 2D and 3D cultures, with the added disease complexity (Figure 2C). Advances in tissue engineering have welcomed the generation of a range of HSE models. This particular *in vitro* HSE model consists of a fibrin-based dermal matrix composed of human KC and FB cultured with a serum-free supplementation of AD-inducing Th2 cytokines (Sriram et al., 2019). Ataç and colleagues developed the use of commercially available skin equivalents using chip-based systems to prolong the viability and maintenance of these models (Ataç et al., 2013). This model was able to generate mechanical forces to replicate the *in vivo* environment including movement of signalling molecules and cell-cell communications (Ataç et al., 2013).

Taking it further for drug testing, an HSE-on-a-chip microfluidic system was designed to allow for up to 3 weeks maintenance of the FT-HSE (Abaci et al., 2015). Another study used HaCaTs as the epidermal layer and co-cultured it with human leukemic monocyte lymphoma cell line (U937) as dendritic cells in a microfluidic system. They induced inflammation by adding LPS and observed cytokine expression. The model studied the regulating role of KC in the context of dermatitis and chemical/biological hazards (Ramadan and Ting, 2015).

In order to further improve these models, a study proposed a vascularised model consisting of epidermal, dermal and endothelial layers, separated by a porous membrane, and then inducing inflammation by adding TNF-α on the dermal layer (Wufuer et al., 2016). Another study developed a perfusable and vascularised 3D-HSE in a 3D-printed mould with the preferred vasculature pattern. This study was the first to incorporate induced Pluripotent Stem Cells (iPSCs)-generated vasculature (Abaci et al., 2016). Ren and colleagues developed another multi-cellular skin-on-a-chip model targeted at studying *trans*-endothelial and *trans*-epithelial migration of T cells in skin inflammation. The chip contained an ECM composed of a type I collagen porous structure with HaCaT cells on one side representing the epidermis, and a HUVEC layer on the other side comprising the endothelium. The authors demonstrated the potential application of this model to investigate T cell transmigration in response to inflammation or drugs by adding TNF-α into the HaCaT layer, and T cells were shown to migrate from the HUVEC layer across the collagen ECM layer and towards HaCaTs (Ren et al., 2021). Table 1 summarizes some of the *in vitro* disease models available to study PS and AD.

**TABLE 1:** A summary of *in vitro* 2D and 3D disease models available to study psoriasis and atopic dermatitis.

Model	Matrix	Disorder	Cellular components	Morphological hallmarks	Measured markers	Ref
2D models						
Co-culture + immune cells		PS	Psoriatic KC and healthy T cells	N/A	**↑** TNF-a, IL-6, GM-CSF, IL-8, MCP-1 and IP-10	Martin et al. (2012)
		AD	FB + eosinophils/basophils + NOD2/TLR2 ligand	N/A	↑ CXCL8, CCL2, CCL5, IL-6, CCL4	Jiao et al. (2016)
		PS	HaCaT + cytokines: IL-17A, IL-22, IL-1α, TNF-α and oncostatin M	Psoriasiform at transcription level	↑ antimicrobial peptides BD2, S100A7, S100A8, S100A9	Zheng et al. (2021)
					↑ CXCL1, CXCL2, CXCL8, CCL20, IL-1β, IL-6, IL-18	
					↓ mRNA Keratin1, Keratin10, Filaggrin, Loricrin	
3D Models						
RHE	EpiDerm, MatTek	PS	Normal primary human KC	Acanthosis and Hyperkeratosis	↑ mRNA Keratin16, S100A7, CXCL1/8/20, CCL2, DEFB4	Sa et al. (2007)
			Stimulus: IL-19, IL-20, IL-22 and IL-24		↑ protein Keratin16, S100A7, STAT3, pY‐STAT3, IL‐8	
	Collagen model	AD	Epidermis: HaCaT. Stimulus: Activated T cells	Keratinocyte apoptosis	↑ Protein IL-8, NT-4 E-cad, IP-10, TARC, eotaxin	Engelhart et al. (2005)
	Polycarbonate filters	PS	Human primary KC	Parakeratosis, thinner epidermis and compact *stratum corneum*	↑ Lentivirus-mediated shRNA interface ↓ Protein Filaggrin-2 Reduced processing of corneodesmosin and hornerin	Pendaries et al. (2015)
			Filaggrin-2 knockdown using shRNA			
HSE	FT model from MatTek	PS	Epidermis: Healthy KC/Dermis: Healthy FB	Acanthosis and Hyperkeratosis	↑ mRNA DEFB4, CCL20, CXCL8, S100A7	Nograles et al. (2008)
			Stimulus: IL-17 and IL-22			
	DED	PS	Healthy adult KC	Parakeratosis	↑ mRNA SKALP/Elafin, DEFB4	Tjabringa et al. (2008)
			Stimulus: TNF-α, IL-1α, IL-6 and IL-22		↑ protein SKALP/Elafin, hBD2, CK16, TNFα, IL‐8	
					↓ protein CK10	
	Reconstructed Collagen model	PS	Epidermis: healthy/psoriatic KC	Hyperproliferation and parakeratosis	↑ protein TNFα, IFNγ, CXCR2, IL‐8	Barker et al. (2004)
			Dermis: collagen and healthy/psoriatic FB			
	Self-assembly	PS	Epidermis: healthy/Psoriatic KC	Acanthosis, Hyperkeratosis, Hyperproliferation	↑ protein Involucrin	Jean et al. (2009)
			Dermis: healthy/Psoriatic FB		↓ protein Flagirrin, laminin	
	DED	PS/AD	Epidermis: healthy KC	Psoriasiform	↑ mRNA DEFB4, SKALP/elafin, LCE3A, Keratin16, S100A7/8	van den Bogaard et al. (2014)
			Immune stimulus: CD4+ T cells		↑ protein IL‐6, IL‐8, IL‐23, CXCL10, hBD2, CK‐16	
					↓ protein Flaggirin, involucrin	
	FT model, MatTek	PS	Epidermis: Healthy/Psoriatic KC. Dermis: FB	Parakeratosis	↑ mRNA S100A7	Chiricozzi et al. (2014)
			Stimulus: IL-17		↑ protein K16, STAT 3	
	Self-assembly	PS	Epidermis: Healthy KC. DermisFB.	Epidermal acanthosis and hyperproliferation	↑ S100A12, IL-8, DEFB4A, and	Pouliot-Bérubé et al. (2016)
			Stimulus: TNF-α, IL-1α, IL-6 and IL-17A			
Skin-on-a-chip						
RHE	Porous membrane		Epidermis: HaCaTs. Immune component: human leukemic monocyte lymphoma cell line (U937) as dendritic cells + LPS			Ramadan and Ting, (2015)
	Collagen		Epidermis: HaCaT. Endothelial: HUVEC separated by type I Collagen membrane + TNF-α+T cells			Ren et al. (2021)
HSE	Porous membrane		Epidermal (HaCaT), dermal (FB) and endothelial (HUVEC) components. Cytokine TNF-α added			Wufuer et al. (2016)
	EpiDerm		Improved nutritional and cellular components using a flow generator model. The model can apply mechanical stress and extends culture periods			Ataç et al. (2013)
	Porous membrane		Dermal and epidermal components. Keratinocyte cells were used. Maintains HSE for up to 3 weeks in culture			Abaci et al. (2015)
	Collagen		An iPSC generated vascularised 3D HSE in a 3D printed mould with designable vascular patterns			Abaci et al. (2016)
	Collagen		HaCaT/KC + FB + HUVEC + HL 60 cells in SDS and UV irradiation			Kwak et al. (2020)

RHE, reconstructed human equivalent; FT, Full-Thickness; HSE, human skin equivalent; KC, keratinocytes; FB, fibroblasts; HUVEC, human umbilical vascular endothelial cells; PS, psoriasis; AD, atopic dermatitis.

## 3 Discussion

Numerous *in vitro* models for the study of PS and AD pathogenesis have been developed and applied to understand the pathogenesis of skin diseases and to assess for new treatments. Although skin-on-a-chip is a relatively new technology, it seems to be the most promising *in vitro* model for both PS and AD, as a physiologically accurate and controlled environment can be reproduced. Additionally, the model offers improved barrier function and the ability to incorporate different immune cells as well as improved vasculature (van den Broek et al., 2017).

In 2D models, the cellular organisation and interaction can easily be disturbed, affecting cellular cross-talking (Groeber et al., 2011). Additionally, the use of scaffolds in 3D models helps improve the mechanical characteristics of dermal equivalents, by improving their life span, structure, and gene expression (Stark et al., 2006). Another advantage of 3D models is the ability to manipulate their cellular composition according to the types of cells in the model or the cytokines and other components that can be incorporated.

Finally, by developing complex 3D microenvironments, it provides an alternative pre-clinical test for drug screening, which could not be studied using monolayer models. They can provide an alternative to *in vivo* testing which are limited due to the ethical issues as well as the disparity between human and animal responses. Skin-on-a-chip model provides all the advantages of the aforementioned 3D models, with the possibility of mimicking the vascular and molecular environment of the body. This model is also more user-friendly and cost-effective, especially for high throughput drug testing.

## 4 Conclusion and Future Directions

It has become evident that there is not one *in vitro* disease model that is able to fully recapitulate the pathogenesis of either or both diseases, including the molecular aspects. However, with advances in 3D models and the inclusion of immune components, the models have significantly furthered the understanding of these skin diseases thus far, and with applying the appropriate upgrades and technologies the research in this field will be much closer at gaining a fuller understanding of the molecular basis of such inflammatory diseases.

The growing prevalence of these skin diseases has highlighted the increasingly evident unanswered questions and gaps in the field surrounding almost-native models. Skin-on-a-chip is a relatively new concept; however, recent studies have shown that there is a large potential and market for these models particularly in disease modelling, drug discovery and reducing the need for animal trials. Currently the studies have led to the development of predominantly non-vascularised models, with some emerging vascularized models paving the way for future developments of fully vascularised *in vitro* models. Overall, skin-on-a-chip is a promising model to overcome the weaknesses of 2D models, and to further our understanding of the pathogenesis of skin diseases including PS and AD.
